# Poly(ADP-Ribose) Polymerase 1 Promotes Inflammation and Fibrosis in a Mouse Model of Chronic Pancreatitis

**DOI:** 10.3390/ijms22073593

**Published:** 2021-03-30

**Authors:** Tarek El-Hamoly, Zoltán Hajnády, Máté Nagy-Pénzes, Edina Bakondi, Zsolt Regdon, Máté A. Demény, Katalin Kovács, Csaba Hegedűs, Sahar S. Abd El-Rahman, Éva Szabó, József Maléth, Péter Hegyi, László Virág

**Affiliations:** 1 Department of Medical Chemistry, Faculty of Medicine, University of Debrecen, 4032 Debrecen, Hungary; tahamoly@hotmail.com (T.E.-H.); hajnady.zoltan@med.unideb.hu (Z.H.); nagy.mate@med.unideb.hu (M.N.-P.); ebakondi@med.unideb.hu (E.B.); regdon.zsolt@med.unideb.hu (Z.R.); kovacs.katalin@med.unideb.hu (K.K.); hcsaba@med.unideb.hu (C.H.); 2 Drug Radiation Research Department, National Centre for Radiation Research and Technology, Atomic Energy Authority, 11787 Cairo, Egypt; 3 Doctoral School of Molecular Medicine, University of Debrecen, 4032 Debrecen, Hungary; 4 MTA-DE Cell Biology and Signaling Research Group, 4032 Debrecen, Hungary; demenym@med.unideb.hu; 5 Department of Pathology, Faculty of Veterinary Medicine, Cairo University, 12211 Giza, Egypt; saharsamirmah@cu.edu.eg; 6 Department of Dermatology, Faculty of Medicine, University of Debrecen, 4032 Debrecen, Hungary; eszabo@med.unideb.hu; 7 First Department of Medicine, University of Szeged, 6720 Szeged, Hungary; jozsefmaleth1@gmail.com; 8 HAS-USZ Momentum Epithel Cell Signalling and Secretion Research Group, 6720 Szeged, Hungary; 9 Department of Public Health, University of Szeged, 6720 Szeged, Hungary; 10 János Szentágothai Research Centre, Institute for Translational Medicine, Medical School, University of Pécs, 7624 Pécs, Hungary; hegyi2009@gmail.com; 11 Momentum Gastroenterology Multidisciplinary Research Group, Hungarian Academy of Sciences, University of Szeged, 6720 Szeged, Hungary

**Keywords:** chronic pancreatitis, inflammation, fibrosis, cell death, poly(ADP-ribose) polymerase 1

## Abstract

Chronic pancreatitis (CP) is an inflammatory disease of the pancreas characterized by ductal obstructions, tissue fibrosis, atrophy and exocrine and endocrine pancreatic insufficiency. However, our understanding is very limited concerning the disease’s progression from a single acute inflammation, via recurrent acute pancreatitis (AP) and early CP, to the late stage CP. Poly(ADP-ribose) polymerase 1 (PARP1) is a DNA damage sensor enzyme activated mostly by oxidative DNA damage. As a co-activator of inflammatory transcription factors, PARP1 is a central mediator of the inflammatory response and it has also been implicated in acute pancreatitis. Here, we set out to investigate whether PARP1 contributed to the pathogenesis of CP. We found that the clinically used PARP inhibitor olaparib (OLA) had protective effects in a murine model of CP induced by multiple cerulein injections. OLA reduced pancreas atrophy and expression of the inflammatory mediators TNFα and interleukin-6 (IL-6), both in the pancreas and in the lungs. Moreover, there was significantly less fibrosis (Masson’s trichrome staining) in the pancreatic sections of OLA-treated mice compared to the cerulein-only group. mRNA expression of the fibrosis markers TGFβ, smooth muscle actin (SMA), and collagen-1 were markedly reduced by OLA. CP was also induced in PARP1 knockout (KO) mice and their wild-type (WT) counterparts. Inflammation and fibrosis markers showed lower expression in the KO compared to the WT mice. Moreover, reduced granulocyte infiltration (tissue myeloperoxidase activity) and a lower elevation of serum amylase and lipase activity could also be detected in the KO mice. Furthermore, primary acinar cells isolated from KO mice were also protected from cerulein-induced toxicity compared to WT cells. In summary, our data suggest that PARP inhibitors may be promising candidates for repurposing to treat not only acute but chronic pancreatitis as well.

## 1. Introduction

Pancreatitis, an inflammatory disease of the pancreas, affects both the exocrine and endocrine functions of the organ [1]. Pancreatitis is often viewed as a spectrum of conditions ranging from acute to recurrent and chronic pancreatitis (CP) [2]. Whether, when, and how acute pancreatitis (AP) recurs and progresses to CP is mostly unknown [3]. Several key etiological factors have been identified in CP. These include alcohol abuse, smoking, obstruction of the pancreatic duct, nutritional factors and autoimmunity (e.g., systemic lupus erythematosus), but genetic predisposition clearly plays a role in determining disease susceptibility [4]. Advances in the analysis of the genetic background of CP identified mutations and single nucleotide polymorphisms (SNPs) in trypsinogen, protease inhibitors and ion channels. Based on these discoveries, in the last two decades two complementary theories emerged to explain the pathomechanism of CP. One of these focuses on abnormalities of trypsinogen activation as exemplified by gain of function mutations in cationic trypsinogen (PRSS1) [5] or loss of function mutations in the serine protease inhibitor kazal-type 1 (SPINK1) [6]. These mutations lead to enhanced intra-acinar activation of pancreatic proteases, causing autodigestion of pancreatic tissue. On the other hand, impaired fluid transport may prevent dilution of the protein-rich pancreatic juice, resulting in the formation of proteinaceous plugs and, consequently, stones in the ductal and lobular parts of the organ [7,8]. CP-associated mutations in the cystic fibrosis transmembrane conductance regulator (CFTR) channel are in line with this latter theory.

CP may appear in various different forms, but the most common features of CP include fibrosis, acinar atrophy, ductal irregularities, stenosis or dilatation [9]. Histologically, the predominantly mononuclear infiltration also distinguishes CP from AP where granulocytes are the most common inflammatory cells [10,11]. Fibrosis is caused by activation of pancreatic stellate cells (PaSC). In response to stimulation by platelet-derived growth factor (PDGF), PaSCs produce extracellular matrix proteins and deposit them in the pancreatic parenchyma [12].

Poly(ADP-ribosyl)ation (PARylation) has been suggested to contribute to the pathomechanism of AP [13,14,15]. PARylation is a protein modification carried out by some enzymes of the 17-member poly(ADP-ribose) polymerase (PARP) enzyme family. PARP1 is a multifunctional protein that participates in a wide range of biological activities. The best-characterized function of PARP1 is oxidative DNA damage sensing, by which it contributes to the repair of this common type of DNA injury. Interestingly, in the most severe oxidative DNA damage scenarios, an irrepairably high level of DNA damage triggers overactivation of PARP1 resulting in a programmed necrotic cell death modality termed parthanatos. Thus, PARP1 is considered as a molecular switch, making decisions between cell survival and cell death depending on the intensity of the DNA damage [16,17]. Additionally, PARP1 is a co-activator of several inflammatory transcription factors, most notably NFκB and AP1 [18]. A plethora of preclinical studies proved that targeting PARylation is an effective strategy for dampening inflammation e.g., in arthritis, colitis, dermatitis and different forms of shock. Based on this evidence, clinically available PARP inhibitors (PARPi) used for the treatment of BRCA1/2 mutant ovarian and breast cancers have been proposed to be suitable for repurposing to treat inflammatory conditions [19]. Moreover, PARP1 may also be involved in the regulation of extracellular matrix (ECM) production and contributes to fibrosis in models of nephrotoxicity and lipopolysaccharide (LPS)-induced myocardial fibrosis [20,21]. 

Yelamos’s group already showed that PARPi or genetic inactivation of PARP1 attenuates AP and associated lung injury [14,15,22]. This was also confirmed by Ahmad et al. [13]. However, the role of PARP1 and PARylation in CP has not yet been investigated. Since oxidative stress, necrotic cell death and fibrosis are typical characteristics of CP, it seemed plausible to hypothesize that PARylation may be involved in CP pathogenesis. Moreover, since inhibition of PARP activation targets common pathways (expression of inflammation promoting cytokines, chemokines, adhesion factors, inducible nitric oxide synthase, etc.) of various inflammatory conditions, it seemed likely that PARPi could also attenuate CP. Therefore, we set out to examine whether the course of CP can be modulated by targeting PARP1 activation. Our data prove that in a mouse model of CP induced by multiple injections of the secretagogue peptide cerulein, the severity of CP and the associated fibrosis is reduced by the PARP inhibitor olaparib, or when the PARP1 gene is inactivated.

## 2. Results

### 2.1. PARP Inhibition by Olaparib Reduces Tissue Injury in CP

**Figure 1:**
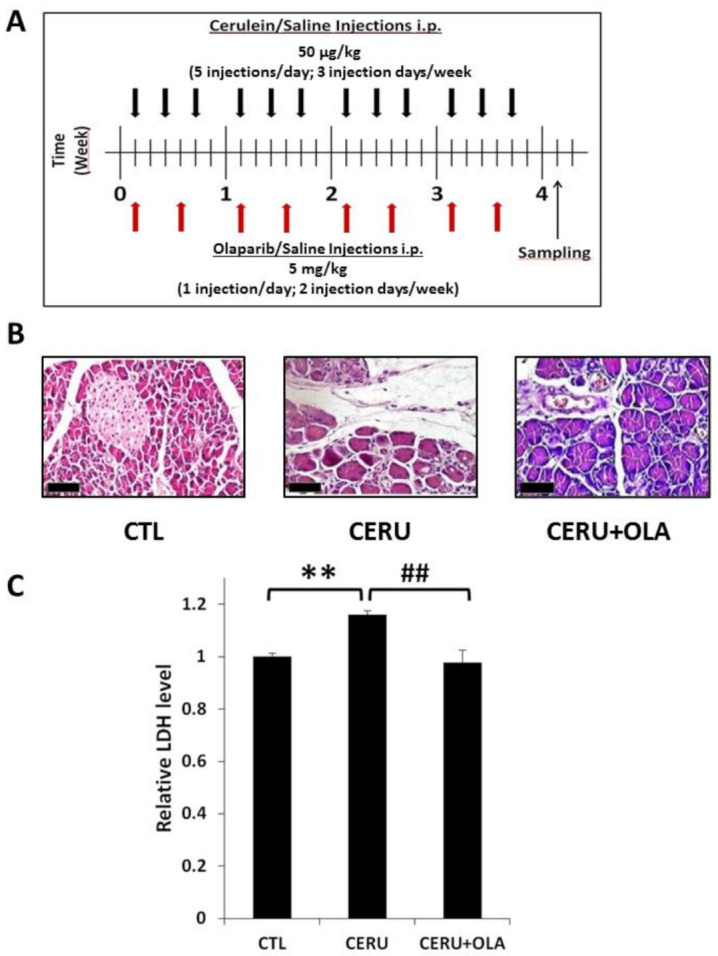
The poly(ADP-ribose) polymerase (PARP) inhibitor olaparib reduces pancreatic injury in cerulein-induced chronic pancreatitis. (**A**) Schematic diagram of the treatment protocol. Black arrows indicate days of cerulein treatment. Olaparib administration (5 mg/kg, twice a week) is indicated with red arrows. Samples were collected 4 days after the last cerulein dose. Mice in the control group were treated with saline. (**B**) Formalin-fixed, paraffin-embedded tissue sections were stained with hematoxylin and eosin. Pictures were taken with a 40 × objective (scale bar 50 µm). Pancreatic tissue of control mice revealed normal histological structure of both endo and exocrine parts, while cerulein-treated mice showed severe histological alterations: interstitial edema, mononuclear inflammatory cell infiltration and marked fibroplasia. The acinar cells showed marked degenerative changes, signs of cell death and lobular atrophy. Mice treated with olaparib showed significant decrease in all these parameters. (**C**) LDH levels were measured in sera collected from all experimental groups. Statistical analysis of experimental data was performed with ANOVA method, followed by Tukey–Kramer test for multiple comparisons. Bars represent SEM for six mice per group. Results considered significant at *p* < 0.01 (**) versus vehicle-treated control, or *p* < 0.01 (^##^) vs. cerulein-treated group.

Prolonged treatment with cerulein, a secretagogue cholecystokynine analogue peptide, is a well-established model of CP. Similarly to published data, we found that repeated doses of cerulein caused pancreatic injury (Figure 1B,C). Cell damage was also indicated by increased serum levels of LDH (Figure 1C). Analysis of the tissue architecture revealed no noticeable changes in the control pancreas section. The pancreata showed normal morphology (Figure 1B). In the CP group, however, signs of tissue edema could be observed. The acinar cells showed marked degenerative changes (granular and vacuolar types). Cells with apoptotic and necrotic morphology were also present. Acinar necrosis and lobular atrophy could be observed. The pancreatic tissue was infiltrated with inflammatory cells and acinar atrophy could be observed (Table 1). Olaparib treatment significantly reduced cerulein-induced LDH release and acinar atrophy (Figure 1B,C). The PARP inhibitor also inhibited inflammatory cell migration into the pancreas (Table 1).

### 2.2. Olaparib Suppresses Expression of Inflammatory Mediators in CP

**Figure 2:**
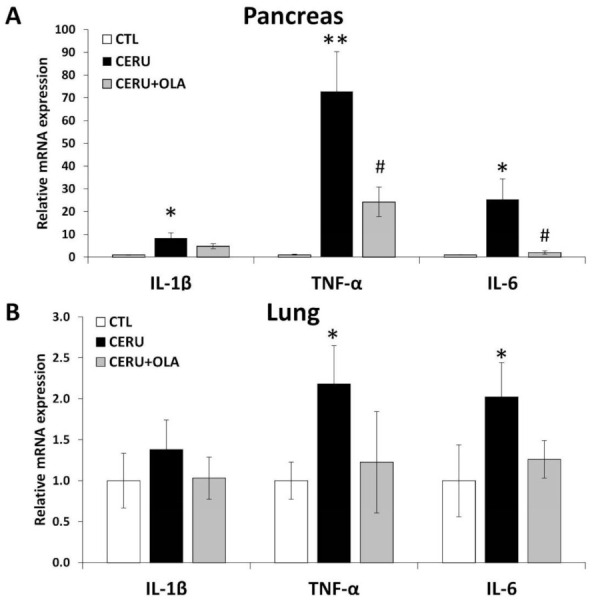
PARP1 activation mediates the inflammatory response in cerulein-induced CP. Olaparib-treated animals showed a decrease in relative expression of inflammatory markers in tissue homogenates of (**A**) pancreases and (**B**) the lungs. Statistical analysis of experimental data was performed with ANOVA, followed by Tukey–Kramer test for multiple comparisons. Error bars represent SEM for *n* = 8. Results considered significant at *p* < 0.05 (*) or *p* < 0.01 (**) vs. vehicle-treated control, or *p* < 0.05 (^#^) vs. cerulein-treated group.

Tissue levels of various inflammatory cytokines such as IL-1β, TNFα and IL-6 were elevated in the pancreases of CP mice (Figure 2A). In line with our current understanding of CP as a systemic disease affecting distant organs, we measured higher TNFα and IL-6 levels in the lungs of CP animals (Figure 2B). Olaparib significantly inhibited TNFα and IL-6 expression in the pancreas (Figure 2A). Similar changes could also be observed in the lungs without reaching statistical significance (Figure 2B). 

### 2.3. Olaparib Suppresses CP-Associated Pancreatic Fibrosis

**Figure 3:**
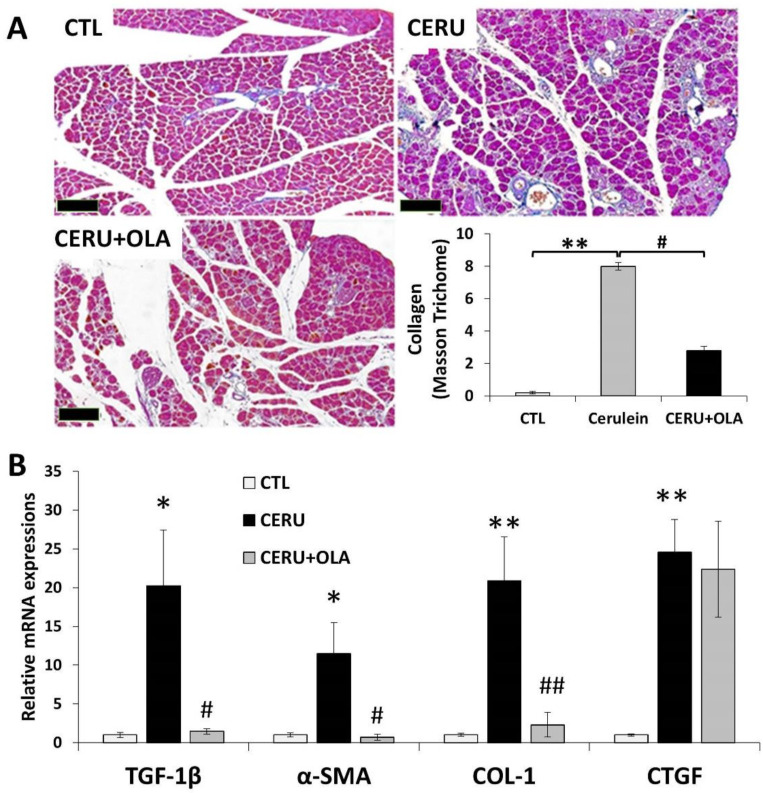
Olaparib inhibits fibrosis in chronic pancreatitis. Mice were treated with either repeated doses of cerulein or cerulein + olaparib for four consecutive weeks (for details see the Materials and Methods section). (**A**) Pancreata were removed, formalin-fixed and stained with Masson’s stain. The percent area covered by collagen fibers was quantified with ImageJ for five separate microscopic fields (5×). Images were taken with 5 × objective (scale bar 200 µm. (**B**) Expression of a set of fibrosis biomarkers (TGF-β1, α-SMA, CTGF and COL-1) was determined with quantitative re-al-time reverse transcriptase PCR (qRT-PCR). Statistical analysis of experimental data was performed with ANOVA method, followed by the Tukey–Kramer test for multiple comparisons. Bars represent SEM of three independent samples; *p* < 0.05 (*) or *p* < 0.01 (**) vs. vehicle-treated control, or *p* < 0.05 (^#^) or *p* < 0.01 (^##^) vs. cerulein-treated group

Tissue fibrosis is the most characteristic feature of chronic inflammations including CP. In our CP model, Masson’s trichrome staining revealed pancreatic collagen deposition indicative of tissue fibrosis (Figure 3A). Perilobular, interlobular, periductal and pericellular fibrosis were apparent. Olaparib reduced collagen deposition as confirmed by semiquantitative evaluation of the staining (Figure 3A). Increased mRNA expression of the fibrosis-inducing cytokine TGFβ and fibrosis markers (collagen I, αSMA and CTGF) could be observed in CP samples. Olaparib improved pancreatic fibrosis scores (Table 1) and significantly reduced mRNA expression of TGFβ, collagen I and αSMA (Figure 3B). The latter could also be confirmed at the protein level (not shown). Overall, these data indicate that PARylation is a mediator of tissue fibrosis in CP.

### 2.4. Reduced Pancreatic Injury and Fibrosis in PARP1 Knockout Animals

**Figure 4:**
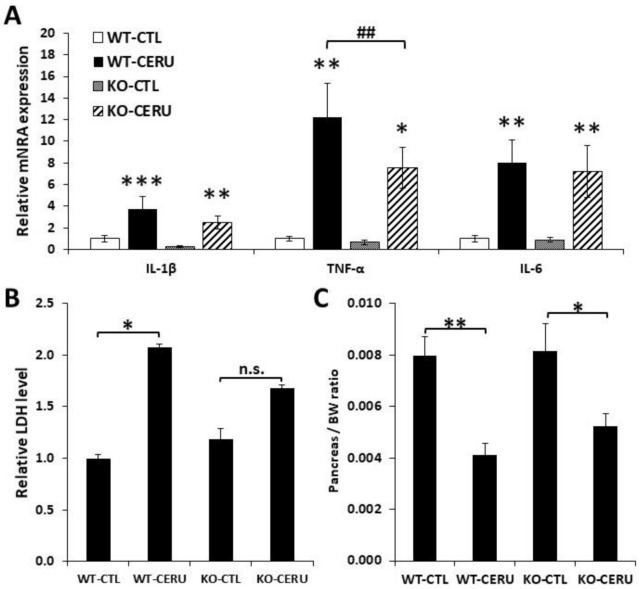
Active role of PARP1 in cerulein-induced chronic pancreatitis. Wild-type (WT) and PARP1 knockout (KO) mice were i.p. treated with cerulein (50 µg/kg, 5 h/3 days/week) for four consecutive weeks as described in the Materials and Methods section. (**A**) Relative mRNA expression of proinflammatory cytokines (IL-1β, TNFα and IL-6) were determined in pancreatic tissue homogenates from cerulein-treated (CERU) or untreated (CTL) mice. Fold ex-pression was normalized to the housekeeping GADPH gene expression. Values from different groups were compared with WT control. (**B**) Serum LDH levels were measured. (**C**) Ceru-lein-treated PARP1 knockout mice showed a moderate decrease in pancreas/total body weight ratio compared to wild-type mice. Error bars represent SEM for *n =* 6. Results considered significant at *p* < 0.05 (*), *p* < 0.01 (**) or *p* < 0.01 (***) vs. vehicle-treated control (ANOVA method, followed by Dunnett’s test for multiple comparisons). *p* < 0.01 (^##^) vs. cerulein-treated group (Student *t* test).

The CP model was also set up with PARP1 knockout (KO) animals and their wild-type (WT) littermates (Figure 4). CP was less severe in PARP1 knockout animals as indicated by moderate acinar atrophy and inflammatory cell migration (Table 2, Supplementary Figure S1). LDH release and pancreas weight reduction indicated pancreas injury/atrophy and both parameters were clearly but non-significantly lower in the KO group compared to the WT group (Figure 4 B,C). Somewhat surprisingly, only IL1β but not TNFα or IL6 showed a significantly lower mRNA expression level in KO animals compared to the WT group (Figure 4A). 

**Figure 5:**
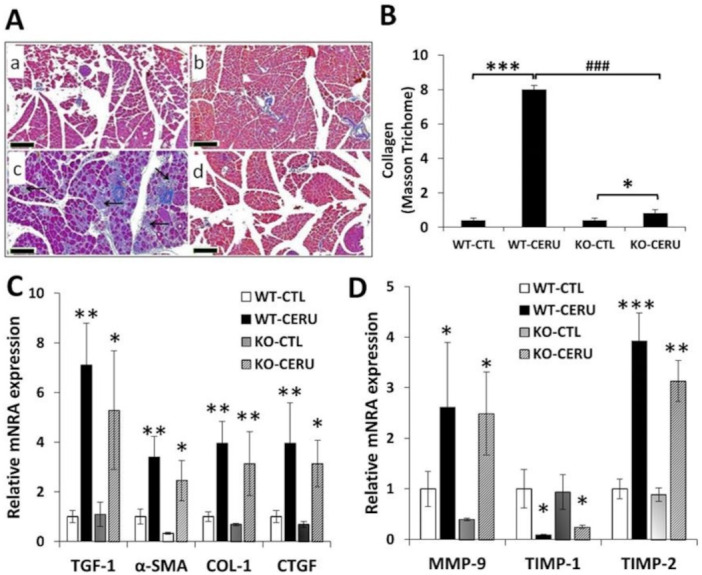
PARP1 mediates pancreas fibrosis in chronic pancreatitis. Wild-type (WT) and PARP1 knockout (KO) mice were intraperitoneally treated with cerulein (50 µg/kg, 5 h/3 days/week) for four consecutive weeks as described in the Materials and Methods section. Pancreata were formalin-fixed and stained with Masson’s stain. The collagen-covered area was quantified by ImageJ for five separate microscopic fields (5×) (**A**,**B**). Images of tissues from WT-CTL and KO-CTL (taken with 5× objective, scale bar 200 µm) showed no pathological alterations (**A**,**B**). Increased periductal, inter- and intra-lobular and pericellular fibrosis could be detected in the WT-CERU mice (**C**) compared to KO-CERU group (**D**). mRNA expression of fibrosis biomarkers (**C**) as well as matrix metalloproteinase-9 (MMP-9) and its tissue inhibitors (TIMP-1 and TIMP-2) (**D**) were determined with qRT-PCR. Data are presented as mean ± SEM. Results considered significant at *p* < 0.05 (*), *p* < 0.01 (**) or *p* < 0.01 (***) vs. vehicle-treated control (ANOVA method, followed by Dunnett’s test for multiple comparisons). *p* < 0.001 (^###^) vs. cerulein-treated WT group (Student *t* test).

Masson’s trichrome staining revealed extensive fibrosis in the WT-CERU group whereas CP-induced collagen deposition was markedly reduced in the pancreata of knockout animals (Figure 5A,B and Table 2). mRNA expression of fibrotic mediators tended to be lower in the KO-CERU group than in the WT-CERU group (Figure 5C,D) without reaching statistical significance. 

### 2.5. Reduced Acinar Cell Damage/Death and Granulocyte Infiltration in PARP1 Knockout Animals

To confirm acinar protection in PARP1 knockout mice, we also tested acinar injury in the simpler acute disease setting. In this model of acute pancreatitis (AP), cerulein was administered on a single day (in eight hourly injections). The lower cerulein-induced serum amylase and lipase levels in the KO mice reflected reduced acinar cell injury in the absence of PARP1. Similarly, MPO levels were also lower in the KO mice indicating an active role of PARP1 in inflammatory cell recruitment to the pancreas.

**Figure 6:**
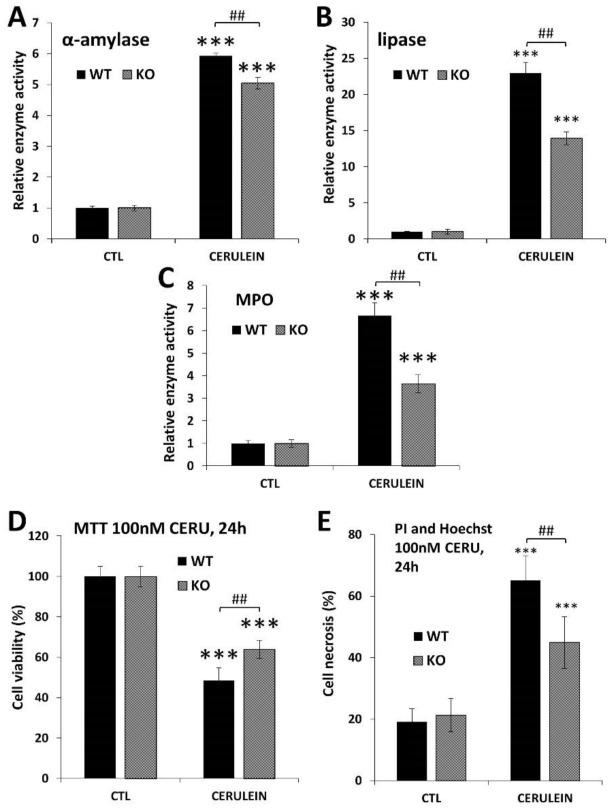
Suppression of acinar cell injury, cell death and granulocyte infiltration in PARP1 knockout mice (**A**–**C**) and acinar cells (**D**,**E**). Data are presented as mean ± SEM (*n* = 6). Results considered significant at *p* < 0.001 (***) vs. vehicle-treated control (ANOVA method, followed by Dunnett’s test for multiple comparisons). Hashmarks indicate significant (^##^ *p* < 0.01 difference between cerulein-treated WT and KO groups (Student-*t* test).

To prove that PARP1 activation is involved in cerulein-induced acinar cell damage, we isolated primary acinar cells from WT and KO mice and treated the cells in vitro with cerulein. Our data show that KO acinar cells were protected from cerulein-induced loss of viability (Figure 6D). More specifically, propidium iodide uptake assays revealed that cerulein caused necrosis in isolated acinar cells. Cells isolated from PARP1 knockout mice proved resistant to cerulein-induced necrotic cell death (Figure 6E).

Wild-type (WT) and PARP1 knockout (KO) mice were treated with cerulein to induce acute pancreatitis according to the reference given in the Materials and Methods section. (A,B) α-amylase and lipase levels were determined in cerulein-treated (CERU) and untreated (CTL) mice. Cerulein induced less amylase and lipase induction in the PARP1 KO mice compared to the WT controls. (C) KO mice also showed lower pancreatic MPO levels (indicator of neutrophil infiltration) compared to WT mice. (D) Viability of isolated acinar cells decreased 24 h after cerulein (100nM) treatment but cells isolated from PARP1 KO mice were resistant to the damage as assessed in MTT assay. (E) Cerulein-induced necrosis was quantified after double staining of isolated acinar cells with propidium iodide and Hoechst dye. High-content analysis revealed suppressed necrosis in the KO cells compared to WT ones.

## 3. Discussion

Chronic pancreatitis is an inflammatory disease of the pancreas affecting mainly the exocrine function of the organ. Pancreatitis has a complex etiology and a pathomechanism that involves alcohol abuse, cigarette smoking and hereditary conditions. Repeated episodes of acute inflammation accompanied by acinar cell necrosis and impaired ductal secretion leads to progressive damage of the exocrine pancreas [2,23]. In turn, fibrotic tissue replaces normal parenchyma and exocrine pancreatic insufficiency develops. Currently, there is no definitive treatment for this chronic disease that significantly impairs the patients’ quality of life due to malnutrition, diarrhea and chronic abdominal pain [24].

PARP1 has previously been implicated in the pathomechanism of AP and PARPis have been proposed to be effective in the treatment of AP. Indeed, PARPi can block the vicious cycles of AP pathogenesis acting in various key steps. On the one hand, PARPis provide protection from oxidative-stress-induced and PARP1-mediated necrotic cell death, termed parthanatos. On the other hand, PARPi can disrupt inflammatory signaling orchestrated by inflammatory transcription factors, such as NFκB, and AP1. These cytoprotective and anti-inflammatory effects of PARPis likely contribute to their protective effects in AP. Moreover, PARP1 knockout mice are also protected from AP, proving the involvement of PARP1 in the pathogenesis of AP. However, the role of PARP1 and PARylation has not yet been investigated in chronic pancreatitis.

In our study, pancreatic overstimulation with repeated injections of the cholecystokynine analogue peptide cerulein was used to model CP. To investigate the role of PARylation in CP, the clinically used PARPi olaparib was administered to one group of mice. Similar to its protective effect in AP [13], olaparib also provided protection from CP. Although serum levels of the cell injury marker enzyme LDH were not as dramatic in CP as in AP, olaparib abolished the CP-associated elevation of serum LDH levels. Moreover, inflammatory cytokine mRNA levels were elevated in the CP group, but olaparib treatment significantly suppressed the expression of IL1β, TNFα and IL6. This effect is in line with the co-activator role of PARP1 in the NFκB and AP1-driven upregulation of inflammatory mediators. Pancreatitis is often associated with respiratory failure (lung injury) with the involvement of all of these cytokines [25]. Thus, from the clinical point of view, it may be of high interest that olaparib also appeared to reduce inflammatory cytokine expression in the lungs (even if these clear trends did not reach a statistically significant level). 

Compared to AP, one of the distinguishing features of CP is tissue fibrosis [26]. Masson’s trichrome staining clearly revealed CP-associated collagen deposition, indicating fibrosis. There was markedly reduced fibrosis in the pancreases of animals that received PARPi. Moreover, fibrosis marker genes were also upregulated in CP, but much less so in the olaparib-treated group. In other settings unrelated to CP, it has previously been demonstrated that PARP1 regulates collagen production and fibrosis. For example, PARP activation has been demonstrated, or PARylation has been implicated, in collagen production/fibrosis in the kidneys of cholestatic rats [21], in the heart and lungs of mice with LPS-induced myocardial or pulmonary fibrosis [20,27], in the liver of carbon tetrachloride-treated mice [28], in bleomycin-induced pulmonary fibrosis [29] and in angiotensin II-induced aorta fibrosis [30]. However, the role of PARP1 in CP-associated fibrosis was first demonstrated in the current paper. As for the molecular links connecting PARP1 to fibrosis, they may include both the TGFβ-SMAD pathway and NFκB signaling. TGFβ is the master regulator of fibrosis and it triggers PARylation of SMAD3, enhancing DNA binding of the transcription factor [31]. Suppression of TGFβ-SMAD signaling has been proposed to underlie the antifibrotic effects of PARP inhibition in some of the above models [32]. TGFβ also activates NFκB, and this axis has been implicated in pancreatic stellate cell activation in CP. Considering the co-activator role of PARP1 in NFκB signaling, this connection may also be relevant in the antifibrotic effect of PARP inhibition.

Another line of investigation in our current study involved PARP1 knockout mice. Comparing CP in wild-type and knockout animals confirmed the active role of PARP1 in the pathomechanism of CP. Tissue injury, inflammation and fibrosis were all milder in the knockout mice compared to their wild-type counterparts. Our data also suggest that PARP1 plays an active role in the propagation of acinar cell death. Reduced oxidative stress, milder DNA damage and a mitigated mitochondrial dysfunction have been proposed to be key factors in the cytoprotective effects of PARP inhibition or knockout in various experimental settings. It is quite likely that the same factors are responsible for the reduced cell injury observed here in the PARP1 KO mice. Interestingly, pharmacological PARP inhibition by olaparib appeared to be more effective than knocking out the PARP1 gene. This is in line with previous observations reporting marked protective effects with the PARP inhibitors PJ34 and 3-aminobenzamide in acute pancreatitis [15]. These findings may indicate the involvement of other PARP enzymes in the pathogenesis of AP and CP. The target spectrum of olaparib is rather narrow and involves PARP1-4 [33]. However, unlike PARP1 knockout mice, PARP2 knockouts are not protected from AP [15]. Therefore, PARP2 inhibition by olaparib is not likely to be responsible for the extra protection provided by the PARPi compound compared to the PARP1 KO phenotype. However, the roles of PARP3 and PARP4 cannot be excluded, even if no signs in the current literature suggest such a connection. Future studies assessing the effect of olaparib in PARP1 knockout mice may provide answers regarding the potential roles of PARP3 and PARP4 in CP.

In conclusion, we identified an active role of PARP1 in chronic pancreatitis. The protective effect of PARPi is likely to have a complex mechanism combining the cytoprotective, anti-inflammatory and antifibrotic effects of PARP inhibition. Our data suggest that targeting PARP1 may represent a novel therapeutic modality in CP. Whether or not promising results in this animal study translate to human settings remains to be seen. From the translational perspective, it is particularly encouraging that in the current study the protective effect has been obtained with a drug already approved for oncological applications. Our data suggest that repurposing olaparib for the treatment of acute and chronic pancreatitis may represent a novel therapeutic approach in these potentially life-threatening conditions. However, it must be noted that in this study, olaparib administration was started early. Follow up studies are needed to find out if PARPi treatment started after the first few initial cerulein doses also provides therepautic benefit in CP.

## 4. Materials and Methods

### 4.1. Chronic Pancreatitis Model

All animal experiments were approved by the national authority (protocol number: 25/2017/DEMÁB). Adult male C57/BL6 mice (6–8 weeks old) purchased from Charles River Laboratories (Veszprém, Hungary) were used for evaluating the effect of the PARP1 inhibitor olaparib on chronic pancreatitis (CP). The CP model was set up as previously described in [34]. Briefly, mice were challenged with repeated 50 µg/kg intraperitoneal injection of cerulein (MedChem Express, Monmouth Junction, New Jersey, USA) (5 hourly shots/day on days 1, 3, 5, 8, 10, 12, 15, 17, 19, 22, 24 and 26). The treatment protocol is presented in Figure 1A. Cerulein dosage was adjusted to the initial body weight of the animals. When cerulein and olaparib were given on the same day, cerulein was injected first and olaparib was administered between the second and third cerulein doses (half an hour after the second and before the third cerulein dose).

C57/BL6 mice (*n* = 18) were randomly assigned to three experimental groups. Group 1: saline-treated control (CTL) group, Group 2: cerulein-treated CP group and Group 3: cerulein treatment complemented with Olaparib (SeleckChem, Houston, TX, USA) administration (5 mg/kg) twice a week, for 4 consecutive weeks. Since, according to our experience, under the applied settings the body weight of the animals does not change significantly during the course of the model, dosing was calculated based on the initial body weight of the animals.

For another set of pancreatitis experiments, male (6–8 weeks old) homozygous PARP1 knockout mice (KO) and their respective wild-type (WT) littermates were used. PARP1 knockout genotyping was carried out in accordance with a formerly published method [35]. In this experiment, PARP1 WT and KO mice (*n* = 24) were randomized into four groups (WT-CTL, WT-CERU, KO-CTL and KO-CERU) and CP was induced as described above.

Four days after the last cerulein injection, blood samples were collected under gentle isoflurane anesthesia prior to sacrificing the mice. Pancreatic tissue samples were removed and stored at −80 °C for further biochemical analysis. Other sections of pancreases were kept in 8% formalin solution for histology.

Acute pancreatitis was induced exactly as described before [36].

### 4.2. Lactate Dehydrogenase (LDH), Amylase and Lipase Activity Measurements

Injury of the exocrine pancreas was assessed by measuring serum LDH activity. Serum lactate dehydrogenase (LDH) activity was measured with a commercial kit according to the manufacturer’s instructions (G-Biosciences, St. Louis, MI, USA).

### 4.3. H&E and Masson’s Trichrome

H&E stained sections were graded by an expert pathologist unaware of the identity of the samples and the experimental design. Scoring was based on the extent and severity of various histological parameters including edema, inflammatory cell infiltration, acinar necrosis, acinar atrophy and fibrosis. The latter was subdivided into interlobular, intralobular and perilobular fibrosis as presented in Supplementary Table S1. The total histological score was the sum of the combined scores of all histological parameters (inflammatory infiltrate, atrophy and fibrosis) [37]. Collagen deposition was further assessed based on Masson’s trichrome staining (MTS). Fibrous tissue proliferation was quantified in ImageJ by determining the percent MTS-stained area.

### 4.4. Measurement of Cell Viability (MTT Assay) and High-Content Analysis (HCA) Cytotoxicity

Acinar cells were isolated from 10-week-old male PARP1 KO and WT mice. Cells were seeded into CellCarrier Ultra 96 well microplates (Perkin Elmer, Waltham, MA, USA) in a final volume of 100 μL. The plates were incubated in a CO_2_ incubator overnight. Cerulein was added to the wells (final concentration: 100 nM). The MTT viability assay was carried out as described [38]. For HCA analysis, cells were stained with propidium iodide (2.5 μ g/mL) and Hoechst (5 μ g/mL) for 10 min. Images were acquired and analyzed as previously reported [38] with an Opera Phenix HCS system (Perkin Elmer, Waltham, MA, USA) using a 10X air objective and appropriate laser and filter settings in sequential mode to avoid overlapping of the emission spectra.

### 4.5. RNA Extraction and Real-Time Quantitative PCR Analysis

The assay was carried out as previously described [39]. In brief, total RNA from frozen pancreas tissues was isolated with TRIzol reagent (Thermo Fisher Scientific, Waltham, MA, USA) according to the manufacturer’s instructions. The concentration of the isolated RNA was determined spectrophotometrically and 2 ug were reverse transcribed with Applied Biosystem’s High-Capacity cDNA Reverse Transcription Kit according to the manufacturer’s recommendations. The reverse transcription (RT) reaction was performed at 42 °C for 60 min in a mixture of 2 μL RT buffer, 0.8 μL dNTP, 2 μL random primer, 1 μL reverse transcriptase, 10 μL RNA and 4.2 μL diethylpyrocarbonate (DEPC)-treated H2O, followed by heat inactivation at 95 °C for 5 min. The subsequent quantitative PCR reactions were performed in a Roche LightCycler 480 II thermal cycler using SybrGreen and the primers listed in Supplementary Table S2. The thermal profile of the reactions was the following: 94 °C for 5 min followed by 35 cycles of 94 °C for 30 s, 67 °C for 1 min and 74 °C for 1 min.

## Figures and Tables

**Table 1:** Histological evaluation of the effect of olaparib in chronic pancreatis (CP).

	Control	CERU	OLA + CERU
**Inflammatory infiltrate**	0.2 ± 0.2	2.6 ± 0.25	1.4 ± 0.25
**Acinar atrophy**	0.0 ± 0.0	2.4 ± 0.25	0.6 ± 0.25
**Fibrosis (intralobular)**	0.0 ± 0.0	2.6 ± 0.25	0.8 ± 0.37
**Fibrosis (interlobular)**	0.2 ± 0.2	2.8 ± 0.20	1.2 ± 0.20
**Fibrosis (perilobular)**	0.0 ± 0.0	2.6 ± 0.25	0.8 ± 0.20

**Table 2:** Histology scores of CP in wild-type (WT) and PARP1 knockout (KO) mice.

	WT-CTL	WT-CERU	KO-CTL	KO-CERU
**Inflammatory infiltrate**	0.2 ± 0.2	2.6 ± 0.25	0 ± 0.0	0.6 ± 0.25
**Acinar atrophy**	0.0 ± 0.0	2.4 ± 0.25	0.0 ± 0.0	0.2 ± 0.2
**Fibrosis (intralobular)**	0.0 ± 0.0	2.6 ± 0.25	0.0 ± 0.0	0.2 ± 0.2
**Fibrosis (interlobular)**	0.2 ± 0.2	2.8 ± 0.20	0.2 ± 0.20	0.4 ± 0.2
**Fibrosis (perilobular)**	0.2 ± 0.2	2.6 ± 0.25	0.2 ± 0.2	0.2 ± 0.2

## Data Availability

The data presented in this study are openly available in FigShare at https://doi.org/10.6084/m9.figshare.14333117.

## Supplementary Materials

The following are available online at https://www.mdpi.com/article/10.3390/ijms22073593/s1, Figure S1, Table S1 and Table S2.

## Abbreviations

AP	acute pancreatitis
BSA	bovine serum albumin
CERU	Cerulein
CP	chronic pancreatitis
HCA	high-content analysis
LDH	lactate dehydrogenase
MPO	myeloperoxidase
MTT	3-(4,5-dimethylthiazol-2-yl)-2,5-diphenyltetrazolium bromide
OLA	olaparib
PAR	poly(ADP-ribose)
PARP	poly(ADP-ribose) polymerase
PARPi	PARP inhibitor
PARylation	poly(ADP-ribosyl)ation
PI	propidium iodide
SMA	smooth muscle actin
