# Supplementary material for: Poly(ADP-Ribose) Polymerase 1 Promotes Inflammation and Fibrosis in a Mouse Model of Chronic Pancreatitis
Source: Int J Mol Sci. 2021 Mar 30;22(7):3593. doi: 10.3390/ijms22073593 (PMC8037143; doi:10.3390/ijms22073593)
Supplement: Supplementary file 1 [file ijms-22-03593-s001.pdf]

Supplementary Table S1: Histopathologic Scoring System of Chronic Pancreatitis

Histology	Score	Definition
Inflammatory cell infiltration	0	no infiltrate
	1	mild infiltrate
	2	moderate infiltrate
	3	severe infiltrate
Perilobular fibrosis	0	Absent
	1	Mild
	2	Moderate
	3	Severe
Interlobular fibrosis	0	Absent
	1	Fibrosis between 2 and 3 lobules
	2	Fibrosis between < 50% of lobules
	3	Fibrosis between > 50% of lobules
Intralobular fibrosis	0	Absent
	1	Fibrosis limited to 1-2 lobule (s)
	2	Fibrosis in < 50% of lobules
	3	Fibrosis in > 50% of lobules
Acinar atrophy	0	Absent
	1	Focal atrophy in 1-2 lobule (s)
	2	Focal atrophy in < 50% of lobules
	3	Focal atrophy in > 50% of lobules

Supplementary Table S2: Primer sequences used for qPCR assays

Gene	Primer sequence
Rplp0	AGATTCGGGATATGCTGTTGGC<br><br>TCGGGTCCTAGACCAGTGTTC
RPL13A	GAGGTCGGGTGGAAGTACCA<br><br>TGCATCTTGGCCTTTTCCTT
GAPDH	CAAGGTCATCCATGACAACTTTG<br><br>GGCCATCCACAGTCTTCTGG
IL-1β	CAACCAACAAGTGATATTCTCCATG<br><br>GATCCACACTCTCCAGCTGCA
IL-6	GAGGATACCACTCCCAACAGACC<br><br>AAGTGCATCATCGTTGTTCATACA
TNF-α	CATCTTCTCAAAATTCGAGTGACAA<br><br>TGGGAGTAGACAAGGTACAACCC3
PARP-1	GGAGCTGCTCATCTTCAACC<br><br>GCAGTGACATCCCCAGTACA
PARP-2	GGAAGGCGAGTGCTAAATGAA<br><br>AAGGTCTTCACAGAGTCTCGATTG
TIMP-1	ATTCAAGGCTGTGGGAAATG<br><br>AAGAAGCTGCAGGCATTGAT
TIMP-2	CGTTTCTTTGGGGTTTCTGA<br><br>TTTATCACTAACAATATAGACAGCCACTCT
MMP9	CATTCGCGTGGATAAGGAGT<br><br>ACCTGGTTCACCTCATGGTC
α-SMA	GTCCCAGACATCAGGGAGTAA<br><br>TCGGATACTTCAGCGTCAGGA
Col1a1	GCTCCTCTTAGGGGCCACT<br><br>CCACGTCTCACCATTGGGG
CTGF	CAGAGTGGAGCGCCTGTT<br><br>GGATGCACTTTTTGCCCTTCT
TGF-1β	TGACGTCACTGGAGTTGTACGG<br><br>GGTTCATGTCATGGATGGTGC

Suppl. Figure S1

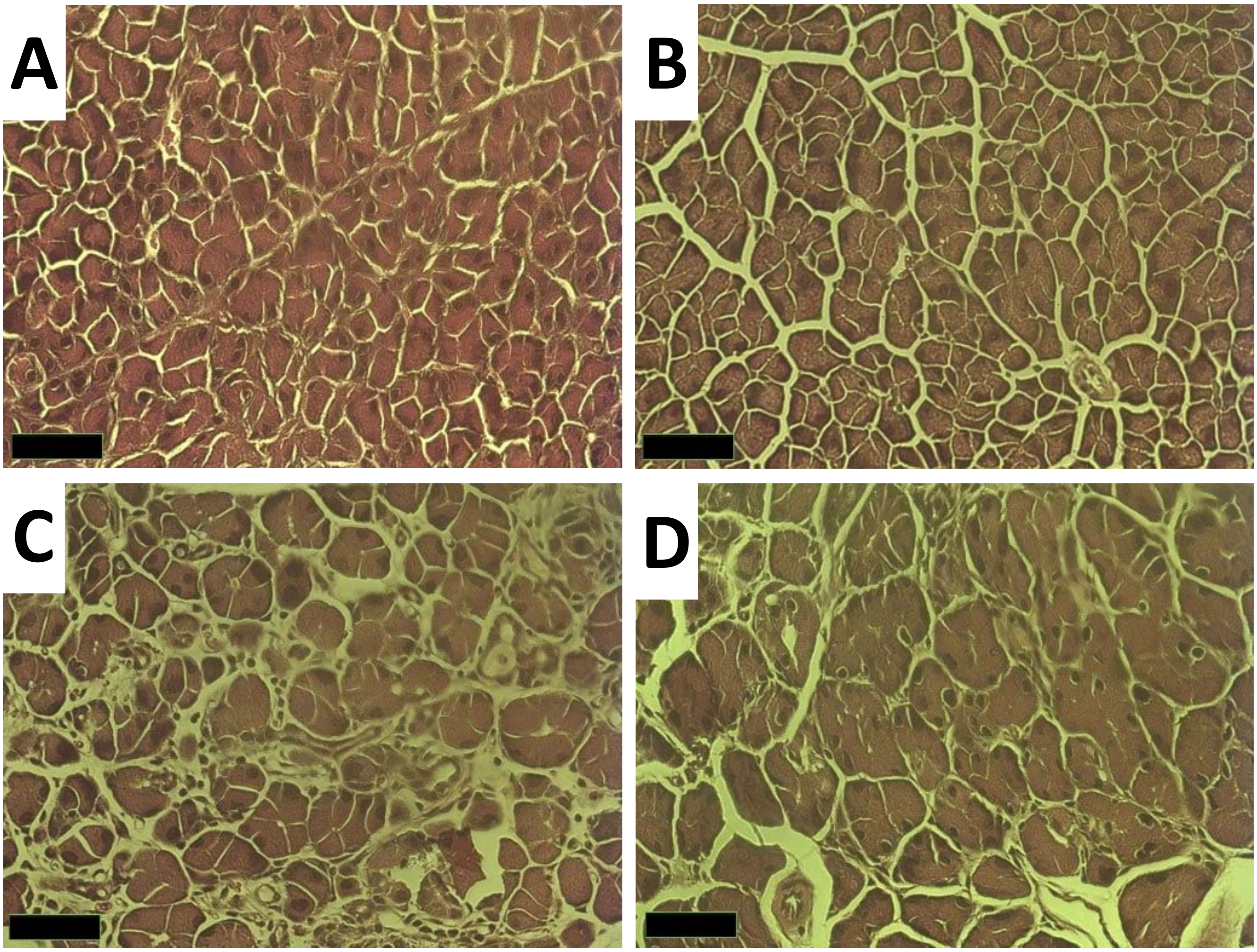

Suppl. Figure S1. Histology of pancreas tissues from wild type and PARP1 deficient mice

Formalin-fixed, paraffin-embedded tissue sections were stained with hematoxylin and eosin. Pancreatic tissue of both wild type (WT) and PARP-1 knockout (KO) mice showed normal exocrine and endocrine tissue architecture (A, B). Cerulein administration to wild type animals induced interlobular edema, mild mononuclear infiltration and marked fibrosis (C). CP induction in KO mice caused only mild fibrosis mostly around the pancreatic ducts without any evidence for inter- or intralobular fibroplasia (D). (Scale bar 50 µm; 40x)
